# Decrease of physical activity level in adolescents with limb fractures: an accelerometry-based activity monitor study

**DOI:** 10.1186/1471-2474-12-87

**Published:** 2011-05-04

**Authors:** Dimitri Ceroni, Xavier Martin, Cécile Delhumeau, Nathalie Farpour-Lambert

**Affiliations:** 1 Pediatric Orthopedic Unit, University of Geneva Hospitals and University of Geneva Faculty of Medicine, Geneva, Switzerland; 2 Clinical Epidemiology Service, University of Geneva Hospitals and University of Geneva Faculty of Medicine, Geneva, Switzerland; 3 Exercise Medicine, Pediatric Cardiology Unit, University of Geneva Hospitals and University of Geneva Faculty of Medicine, Geneva, Switzerland; 4 Department of Child and Adolescent, University of Geneva Hospitals and University of Geneva Faculty of Medicine, Geneva, Switzerland

## Abstract

**Background:**

Immobilization and associated periods of inactivity can cause osteopenia, the physiological response of the bone to disuse. Mechanical loading plays an essential role in maintaining bone integrity. Skeletal fractures represent one cause of reduction of the physical activity (PA) level in adolescents. The purpose of this study was to quantify the reduction of PA in adolescents with limb fractures during the cast immobilization period compared with healthy controls.

**Methods:**

Two hundred twenty adolescents were divided into three groups: those with upper limb fractures (50 cases); lower limb fractures (50 cases); and healthy cases (120 cases). Patients and their healthy peers were matched for gender, age, and seasonal assessment of PA. PA level was assessed during cast immobilization by accelerometer. Time spent in PA in each of the different intensity levels - sedentary, light, moderate, and vigorous - was determined for each participant and expressed in minutes and as a percentage of total valid time.

**Results:**

Reduction in PA during cast immobilization was statistically significant in patients with limb fractures compared to healthy controls. The total PA count (total number of counts/min) was significantly lower in those with upper and lower limb fractures (-30.1% and -62.4%, respectively) compared with healthy controls (p < 0.0001 and p = 0.0003, respectively). Time spent in moderate-to-vigorous PA by patients with upper and lower limb injuries decreased by 36.9% (*p *= 0.0003) and 76.6% (*p *< 0.0001), respectively, and vigorous PA was reduced by 41.4% (*p *= 0.0008) and 84.4% (*p *< 0.0001), respectively.

**Conclusions:**

PA measured by accelerometer is a useful and valid tool to assess the decrease of PA level in adolescents with limb fractures. As cast immobilization and reduced PA are known to induce bone mineral loss, this study provides important information to quantify the decrease of skeletal loading in this patient population. The observed reduction of high intensity skeletal loading due to the decrease in vigorous PA may explain osteopenia due to disuse, and these data should be kept in mind by trauma practitioners to avoid any unnecessary prolongation of the cast immobilization period.

## Background

Bone mineral mass acquired during adolescence is considered as a major determinant of adult bone health [1]. Although genetic factors have a strong influence on peak bone mass, environmental factors, such as physical activity (PA), also contribute [2,3]. Exercise exerts a positive effect on bone growth, particularly if the activity has been initiated before puberty or in the early pubertal period [4-6]. The skeleton appears to be most responsive to mechanical stress during Tanner stages II-IV and corresponds to the two-year window that has been identified as the period of bone mineral accrual during puberty [7,8]. Bone mineral mass is higher in physically active children than in those who are mildly active [9], and higher in children who participate in activities that generate high impact forces than in those who practise activities with lower impact forces [10-12]. Studies have shown that high-intensity forces, especially when imposed during early childhood, produce greater gains in bone mass than low- to moderate-intensity forces [13-18]. Based on this evidence, it is now recommended that physical activity for children include activities generating relatively high ground-reaction forces, such as jumping, skipping, running, and possibly strengthening exercises [5].

It is recognized also that immobilization and associated periods of inactivity can cause osteopenia [19-21], the physiological response of the bone to disuse. Mechanical loading plays an essential role in maintaining bone integrity. Mechanisms underlying bone loss in response to disuse are not yet completely understood, even if it is recognized that they are due to increased bone resorption and corresponding reduced bone formation. In adolescents, loss of bone mineral mass usually occurs during phases of reduced PA, such as cast immobilization [22]. To the best of our knowledge, the measurement of PA levels by accelerometry has not been investigated in adolescents who sustain limb fractures and have to wear a cast for several weeks. The objective of our study was to quantify the reduction of PA levels during cast immobilization in this patient population.

## Methods

### Subjects

One hundred adolescents with a first episode of limb fracture (50 lower limb fractures; 50 upper limb fractures) and a control group of 120 healthy cases were recruited for inclusion in the study through an advertisement for visitors to the Children's Hospital of the University of Geneva Hospitals, Geneva, Switzerland. Exclusion criteria for both injured adolescents and healthy controls were: prior history of bone fractures; chronic disease; congenital or acquired bone disease; any condition limiting physical activity; and hospitalization for more than 2 weeks in the previous 12 months.

Adolescents with limb fractures were treated as inpatients and required general anesthetic for the treatment of their fracture. Those with lower limb fractures received a bent-knee long-leg cast and were instructed to follow a strict zero weight-bearing directive during the initial healing phase. After 3 to 6 weeks, the initial immobilization device was removed and a below-the-knee walking cast was worn until definitive bone healing. All adolescents with upper limb fractures were immoblized into a long-arm cast during the initial healing phase, followed by a forearm cast until definitive bone healing. All study participants and their parents provided written consent and the protocol was approved by the institutional ethics committee (protocol # 04-057, ped 04-002).

### Anthropometric measurements

Standing height was assessed in bare or stocking feet to the nearest 1 mm using a precision mechanical stadiometer (Holtain Ltd, Dyfed, UK). Weight was measured to the nearest 0.1 kg using a mechanical calibrated beam scale (Seca^®^, Reinach, Switzerland). Body mass index (BMI) was calculated as weight (kg)/height squared (m^2^).

### Physical activity measurement

Physical activity was measured during cast immobilization and data were collected from the first day following hospital discharge. Recordings of PA began on a Monday, Tuesday or Wednesday to ensure measurement of at least two weekend days. Objective measurements of PA were obtained using an uniaxial accelerometer (Actigraph^® ^7164, MTI, Fort Walton Beach, FL, USA). The monitor was set on a 1 min cycle and the sum was stored in the memory at the end of each run and the numerical integrator reset. The monitor was attached above the iliac crest of the right hip with an elastic belt and adjustable buckle, and was oriented vertically in the same direction. The accelerometer was programmed to start recording at 8 am on the first day of measurement and participants were asked to wear it continuously, including during the night, for 10 days. Data were collected during all seasons.

### PA data interpretation

Data reduction was based on criteria applied in previous publications [3,23-26]. Only periods between 8 am and 9 pm were analysed. Zero activity periods of 20 min or longer were interpreted as being due to unworn accelerometers and were removed from the activity totals [27]. Participants who did not record more than 600 min/d of activity [3,23-26] for at least 5 days were excluded from further analysis [28]. Data were expressed as total activity counts per registered time (counts/min) to generate an average range of PA. We used the cut-offs of intensity levels described by Ekelund where sedentary behaviour was defined as less than 500 counts/min, light PA from 500 to 1999 counts/min, moderate PA from 2000 to 2999 counts/min, and vigorous PA as > 3000 counts/min [29]. Time spent at each PA intensity level was determined for all participants as a percentage of total valid time.

### Statistical methods

Data are expressed as mean ± SD. A paired Student's t test with an alpha threshold of 5% was used to analyze the variability of matching characteristics (age and gender) for cases and healthy controls. A Shapiro Wilk test with an alpha threshold of 5% was used to test the normality of PA variables. As these variables did not have a normal distribution, a paired Wilcoxon test with an alpha threshold of 5% was used to assess differences of PA levels between cases and healthy controls. Data analyses were performed using STATA 9.2 (StataCorp LP, Texas, USA).

## Results

**Figure 1:**
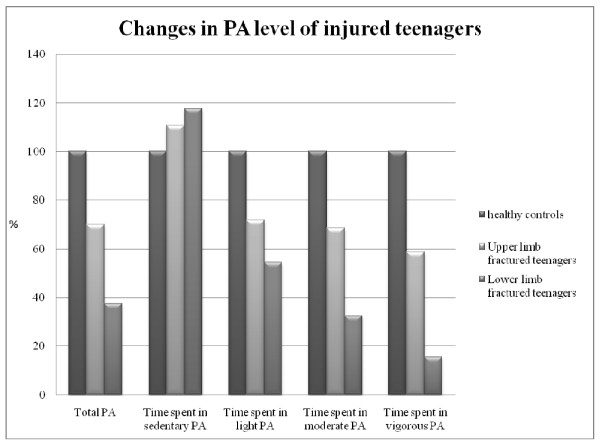
**Physical activity levels of injured teenagers expressed as percentages of healthy teenager's results**. In this graph PA levels of healthy teenagers are 100%. All the differences between injured teenagers and the healthy controls were highly sigificant (For the *p*_values, cf the tables 2 & 3).

Fourteen adolescents with lower limb fractures, 13 with upper limb fractures, and 25 control subjects were excluded either for failing to reach at least 5 days of measurement or instrument malfunction. Thirty-six adolescents with lower limb fractures, 37 with upper limb fractures, and 95 healthy controls were included in the study. Type of fracture, treatment, and cast immobilization duration are shown in Table 1. Patients with limb fractures were paired with healthy controls according to gender, age (± 0.5 years), and seasonal assessment, as the latter may have an impact on the PA level (less sporting activities during the winter months). Thirty-five patients with upper limb fractures were able to be matched with 35 healthy controls; pairing was possible in 34 cases for the lower limb fracture group. Age, physical characteristics, and PA levels of those with fractures and healthy controls are presented in Tables 2 and 3. There was no statistical difference between groups for age, height, weight, BMI, or daily duration of PA monitoring. A statistical difference for the mean number of valid monitored days was observed between patients with lower limb fractures and healthy controls (9.1 ± 1.8 vs 8.2 ± 2, respectively; *p *= 0.028). As expected, cases with limb fractures showed notable reductions in PA levels and spent more time in sedentary activities. The total PA count (number of counts/min) was significantly lower in those with upper and lower limb fractures (-30.1% and -62.4%, respectively) compared with healthy controls. When considering time spent in moderate-to-vigorous PA, a substantial decrease was observed in the upper (-36.9%) and lower limb (-76.6%) fracture groups compared to matched healthy controls. Finally, the decrease in vigorous PA was highly significant for both injured groups (-41.4% and -84.4% for cases with upper and lower limb fractures, respectively) (Figure 1).

**Table 1:** Type of Lesion, Treatment, and Cast Immobilization Duration of Adolescents with Upper and Lower Limb Fractures.

Upper limb (n = 50)		Lower limb (n = 50)	
**Type of lesion**			

Physeal fractures of the wrist	18	Physeal fractures of the ankle	38

Distal metaphyseal fractures of the radius & cubitus of the wrist	29	Distal metaphyseal fractures of tibia and fibula	4

Forearm shaft fractures	3	Leg fractures	4

		Medial malleolus fractures	2

		Lateral malleolus fractures	2

**Treatment **			

Closed reduction and cast immobilization	45	Closed reduction and cast immobilization	33

Stabilization of closed reduction by per-cutaneous wires/screws & cast immobilization	5	Stabilization of closed reduction by per-cutaneous wires/screws & cast immobilization	7

		ORIF and cast immobilization	10

**Cast immobilization duration**			

Long-arm cast immobilization	26.5 +/- 5.8 days	Non-weight-bearing above the knee cast immobilization	27.4 +/- 10.3 days

Forearm cast immobilization	23.3 +/- 10.4 days	Weight-bearing cast	26.4 +/- 8.8 days

The results are expressed as "n" or mean +/- SD

**Table 2:** Characteristics and Physical Activity Measures of Adolescents with Upper Limb Fractures during Cast Immobilization vs Healthy Controls.

	Injured adolescents (n = 35)	Healthy controls (n = 35)	*p *value
Age (yr)	12.6 ± 1.7	12.6 ± 1.8	0.5244

Height (cm)	157.7 ± 13.4	157.7 ± 11.8	0.9989

Weight (kg)	47.3 ± 12.1	46.3 ± 11.4	0.4253

BMI (kg/m^2^)	18.7 ± 2.4	18.4 ± 2.5	0.2610

Number of valid monitored days (days)	8.7 +/- 2.4	8.1 +/- 2.4	0.2044

Daily duration of physical activity monitoring (min)	752.1 ± 26.8	740.9 ± 37.4	0.3177

Total activity (counts/min.day)	417.5 ± 179.1	597.1 ± 211.4	0.0003*

Time spent in sedentary activity (min/day) (% of total daily wearing time)	598.2 +/- 54.7 79.6	540.4 +/- 64.4 72.9	0.0001*

Time spent in light activity (min/day) (% of total daily wearing time)	111.8 +/- 41.1 14.9	136.5 +/- 40.1 18.4	0.0072*

Time spent in moderate (min/day) (% of total daily wearing time)	20.7 +/- 8.4 2.7	30.2 +/- 12 4.1	0.0003*

Time spent in vigorous activity (min/day) (% of total daily wearing time)	21.4 +/- 15.2 2.8	36.5 +/- 18.9 4.9	0.0008*

The results are expressed as mean ± SD.

*: statistically significant difference [please always place at foot of table]

**Table 3:** Characteristics and Physical Activity Measures of Adolescents with Lower Limb Fractures during Cast Immobilization vs Healthy Controls.

	Injured adolescents (n = 34)	Healthy controls (n = 34)	*p *value
Age (yr)	13.6 ± 1.6	13.3 ± 1.6	0.2312

Height (cm)	161.9 ± 11.1	162 ± 12.9	0.9501

Weight (kg)	52.1 ± 11.8	51.8 ± 11.8	0.8128

BMI (kg/m^2^)	19.7 ± 2.5	19.5 ± 2.5	0.2614

Number of valid monitored days (days)	9.1 +/- 1.8	8.2 +/-2	0.0276*

Daily duration of physical activity monitoring (min)	746.7 ± 22.9	749.3 ± 33.3	0.8241

Total activity (counts/min.day)	200.9 ± 92.3	534.5 ± 207.3	<0.0001*

Time spent in sedentary activity (min/day) (% of total daily wearing time)	664.4 +/- 40.9 89	564.3 +/- 64.4 75.3	<0.0001*

Time spent in light activity (min/day) (% of total daily wearing time)	68.4 +/- 26.4 9.2	125.7 +/- 40.1 16.8	<0.0001*

Time spent in moderate (min/day) (% of total daily wearing time)	8.9 +/- 7.2 1.2	27.5 +/- 11.4 3.7	<0.0001*

Time spent in vigorous activity (min/day) (% of total daily wearing time)	5 +/- 6.8 0.6	31.8 +/- 18 4.2	<0.0001*

The results are expressed as mean ± SD.

*: statistically significant difference (please always place at foot of table)

## Discussion

Skeletal fractures represent one cause of reduction of the PA level in adolescents. To the best of our knowledge, this is the first study to report PA measures in a representative sample of this population with upper and lower limb fractures. As expected, we observed a significant reduction of PA levels among injured subjects during cast immobilization, especially those with lower limb fractures. Using total activity measurement, subjects with upper or lower limb fractures were considerably less active (-30.1% and -62.4%, respectively) than healthy fracture-free controls. This was even more marked for time spent in activities of moderate-to- vigorous intensity. For both groups of healthy control subjects, the average time spent in moderate-to-vigorous intensity activity was 59.4 min and 66.7 min daily, whereas it was 42.1 min and 13.9 min, respectively, for adolescents with upper and lower limb fractures.

International guidelines recommend that school-age children should participate daily in at least 60 min of moderate-to-vigorous intensity activity as this level has been shown to prevent weight gain, premature development of cardiovascular diseases and type 2 diabetes, and to increase bone mineral mass [30]. PA reduction in adolescents with fractures results in a decrease in energy expenditure and our clinical experience has shown that this decrease during the cast immobilization period may be the starting point of weight gain for children and adolescents [31].

An examination of time spent in vigorous PA is even more relevant as bone health is related to ground reaction forces [32]. It is well known that an intensification of high impact and weight-bearing exercises has direct and positive consequences in relation to bone mass. Recent studies focus on the theory that high-intensity forces, especially if imposed rapidly, produce greater gains in bone mineral mass than low-to-moderate intensity forces [13,16-18]. Our study investigated specifically the time spent in vigorous PA, which reflects high-intensity forces beneficial to skeletal health, and demonstrated a 41.4% and 84.4% decrease, respectively, in adolescents with upper and lower limb fractures. The significant reduction in high-intensity forces applied to the skeleton may provide also a valid explanation for disuse osteopenia [33]. Restoration of bone mineral mass may occur upon reaching pubertal maturity [33,34] and, above all, normal activity [35]. Nevertheless, there are currently no reported studies on the recovery of PA levels following limb fractures in children and adolescents.

The results of this study constitute a starting point for new investigations. The relationship between PA decrease and bone mineral loss in youth with fractures remains to be established in a prospective study. There are also some limitations to be considered when interpreting the findings of our study. First, our recruitment was relatively heterogeneous, in particular with regard to the practice of sport. Even if we are unable to completely rule out the possibility of selection bias, there is no clear reason to believe that adolescents with markedly different PA profiles would have chosen to participate in the study. Second, there are activities during which accelerometers have to be removed (e.g., swimming) or do not accurately measure the intensity level (e.g., cycling). These "unmonitored" activities may result in an underestimation of PA in healthy controls. Nevertheless, Trost et al reported that children's self-reported periods of "unmonitored" activity added to the registered accelerometer data led to no significant changes in calculated PA levels [36]. Third, to obtain 10 days of measurement recording, activity counts were averaged using a 1-min epoch to ensure that the accelerometer memory capacity was not exceeded. However, this method underestimates vigorous PA as such activity is rarely sustained for longer than 1 min [25]. Although previous studies have demonstrated that vigorous PA may be substantially underestimated [37], this is unlikely to be of importance in this study as we can hypothesize that it will be the same for the different groups.

## Conclusions

PA measured by accelerometer is a useful and valid tool to assess the decrease of PA levels in adolescents with limb fractures. As cast immobilization and reduced PA are known to induce bone mineral loss, this study provides important information to quantify the decrease of skeletal loading in adolescents with limb fractures. The significant reduction in time spent in vigorous PA, which reflects high-intensity forces beneficial to skeletal health, provides a valid explanation for disuse osteopenia. These data are important and should be kept in mind by trauma practitioners to avoid an unnecessary prolongation of the cast immobilization period. This study gives an outline of the decrease of PA due to a sedentary behaviour and emphasizes the role of physical activity. Further research is needed to establish the relationship between the PA level and bone mineral acquisition during cast immobilization and after bone healing.

## Competing interests

The authors declare that they have no competing interests.

## Authors' contributions

DC: participated in the design of the study, conceived and coordinated the study, collected and treated the patients, performed the CSA analysis, and drafted the manuscript. XM: conceived the study, participated in its coordination, collected the data, and performed the statistical analysis. CD: performed the statistical analysis and drafted the manuscript. NFL: participated in the design and conception of the study, and drafted the manuscript. All authors read and approved the final version of the manuscript.

## Pre-publication history

The pre-publication history for this paper can be accessed here:

http://www.biomedcentral.com/1471-2474/12/87/prepub
